# Acquired Bronchobiliary Fistula After a Living Donor Liver Transplant: A Unique Diagnostic and Therapeutic Challenge

**DOI:** 10.14309/crj.0000000000001928

**Published:** 2025-12-12

**Authors:** Olivia Becker, Deepika Suresh, Mary McGrath, Hannah Laird, Curtis Argo, Neeral Shah

**Affiliations:** 1 Department of Internal Medicine, University of Virginia Health, Charlottesville, VA; 2 Division of Gastroenterology and Hepatology, University of Virginia Health, Charlottesville, VA

**Keywords:** bronchobiliary fistula, liver transplant, biloptysis, hepatobiliary iminodiacetic acid scan, endoscopic retrograde cholangiopancreatography

## Abstract

Bronchobiliary fistula (BBF) is an atypical passageway between the biliary system and the bronchial tree. There are scarce reports of BBF and even fewer in living donor liver transplant (LDLT) recipients. We present a case of BBF in a 36-year-old woman who developed a bilious cough 2 years after a LDLT that was complicated by a late bile leak. We conclude that BBF should be considered in LDLT recipients who develop a bile leak postoperatively, given this common risk factor among reviewed cases. We support the use of hepatobiliary scintigraphy for diagnosis and noninvasive approaches aimed at decompression for repair.

## INTRODUCTION

**Figure 1.**
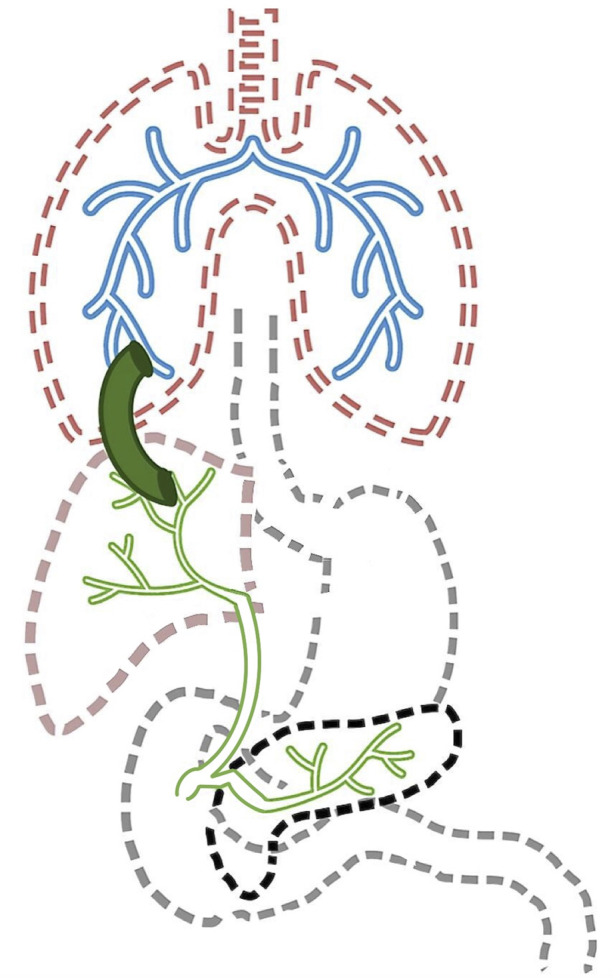
Schematic demonstrating bronchobiliary fistula (connection between biliary system and bronchial tree).

Bronchobiliary fistula (BBF) is a rare phenomenon involving an abnormal connection between the biliary tract and bronchi (Figure 1), which often manifests with biloptysis, or the presence of bile in the sputum. BBF can be both congenital and acquired. Etiologies of acquired BBF include infection, trauma, posthepatectomy complications, biliary tract obstruction, and neoplasm.^1^ This is a case of acquired BBF occurring as a late complication of living donor liver transplant (LDLT) following a bile leak managed with multiple sessions of endoscopic biliary stent placement. Our case adds to the growing discussion regarding workup and management of BBF after a bile leak.

## CASE REPORT

A 36-year old woman presented to a community hospital with fevers and progressive bilious cough (biloptysis) over the last 2 years. Her history included cryptogenic cirrhosis, for which she received a LDLT (right lobe) 4 years before presentation. There were no intraoperative complications. The donor graft had 2 bile ducts in the same lumen, which was anastomosed end to end to the recipient common bile duct. The patient developed a bile leak 4 months postoperatively. She required multiple sessions of biliary stent placement by endoscopic retrograde cholangiopancreatography (ERCP).

**Figure 2.**
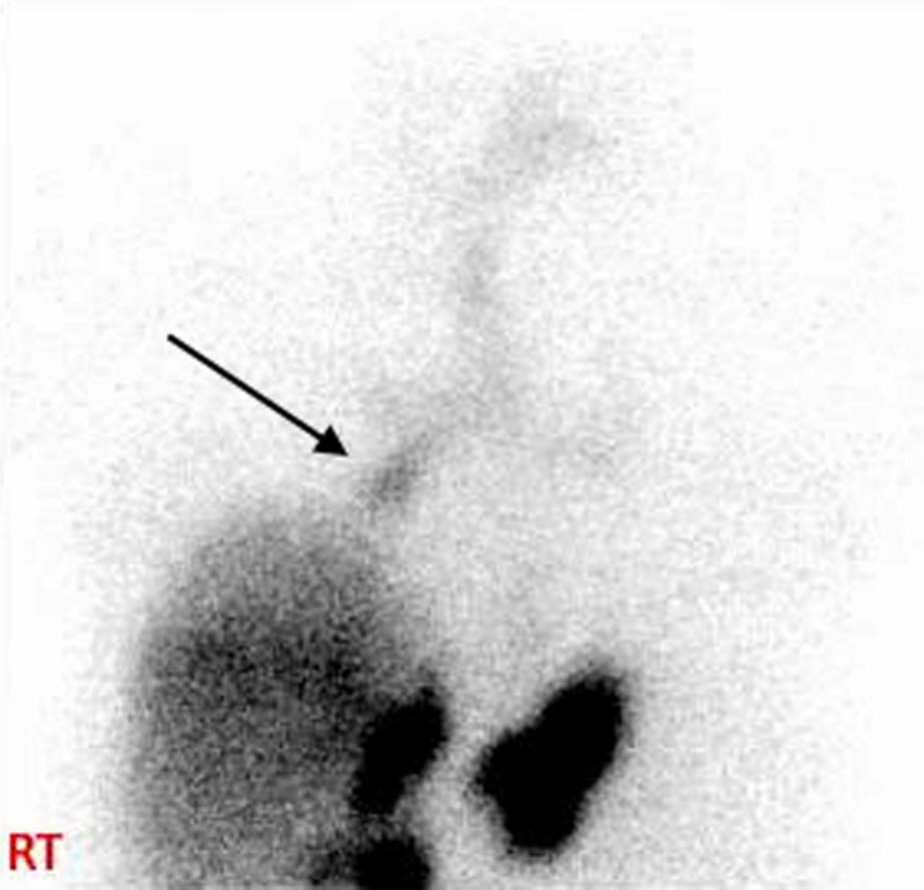
Hepatobiliary iminodiacetic acid scan showing radiotracer uptake extending from the liver dome to the right mainstem bronchus (black arrow) and trachea.

On arrival, she was afebrile but mildly hypotensive. Laboratory studies revealed total bilirubin elevation and elevated alkaline phosphatase. An abdominopelvic computed tomography demonstrated a postoperative fluid collection and locules of gas in the gallbladder fossa and adjacent to the diaphragm and a small right hydrothorax. A hepatobiliary iminodiacetic acid (HIDA) scan demonstrated radiotracer uptake extending from the liver dome to the right mainstem bronchus and trachea (Figure 2), confirming a BBF.

**Figure 3.**
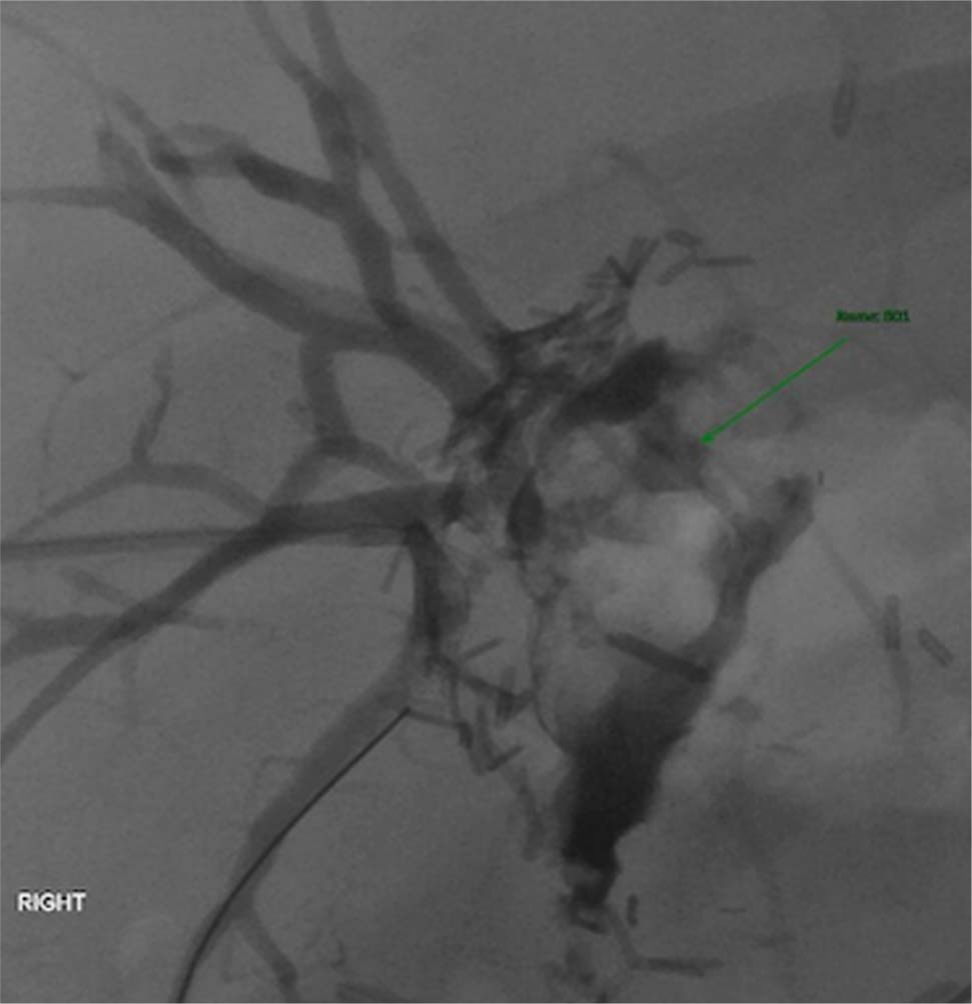
Percutaneous cholangiogram demonstrating a perianastomotic leak (green arrow) at the hepatic hilum.

She was then transferred to an academic center. HIDA scan was not repeated given high clinical suspicion for BBF. She underwent ERCP, which revealed a cavity at the hepatic hilum communicating with the transplanted liver bile ducts. After failed cannulation of the biliary ducts with wire, a biliary stent was placed for percutaneous transhepatic biliary drain targeting. Interventional radiology performed a percutaneous cholangiogram, which revealed a perianastomotic leak at the hepatic hilum without a clearly visualized BBF (Figure 3). A percutaneous biliary drain with custom cut side holes was placed in the right biliary duct, and the patient was discharged after near-immediate improvement of the biloptysis.

**Figure 4.**
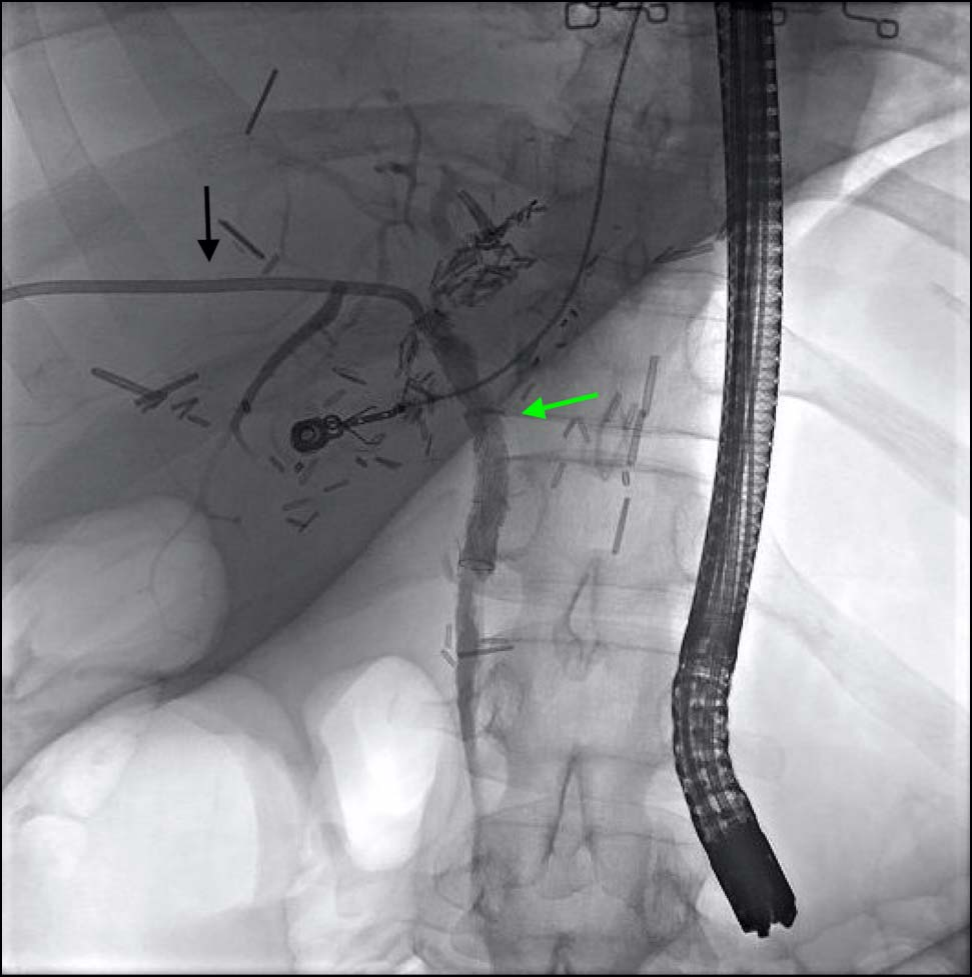
Cholangiogram demonstrating placement of fully covered stent from the right hepatic duct to the mid common bile duct (green arrow) and external drainage catheter (black arrow).

The patient underwent repeat, uncomplicated ERCP with successful biliary access using a rendezvous approach. A fully covered stent was placed from the right hepatic duct to the midcommon bile duct, and interventional radiology exchanged her right internal-external biliary catheter for an external drainage catheter (Figure 4). Repeat cholangiogram demonstrated successful passage of contrast into the bowel through her patent stent, and her external drainage catheter was removed with the hope that her biloptysis will remain controlled.

## DISCUSSION

There are few reported cases of acquired BBF, and to our knowledge, this is the third reported case related to LDLT.^2,3^ Neoplasm and liver surgery complications are the most common etiologies of acquired BBF.^1,4^ Given the paucity of BBF cases, it is essential to report the unique diagnostic and therapeutic approaches of each presentation to guide management of future patients with this condition.

The other cases of BBF after LDLT were diagnosed with magnetic resonance cholangiography, fistulography, and bronchoscopy. One resolved with external and percutaneous transhepatic cholangial drainage after a failed laparoscopic surgical attempt, and the other resolved after external biliary drainage.^2,3^ Postsurgical complications of both cases included a biliary stricture and resultant bile leak. Biliary complications are more common after right-lobe living-donor transplantation, with one review citing a complication rate of 17.1% for bile leaks and 15.2% for biliary strictures.^5^ Transecting the liver exposes numerous bile ducts that become predisposed to leakage.^2,5^ Anatomical features such as small biliary ductal diameter, multiple bile duct openings, and differential blood supply to the bile ducts also increase the complication risk.^6–9^ It is postulated that biliary collections become infected and cause inflammation, thereby forming a tract through necrotic tissue in the neighboring diaphragm. A pressure gradient between the intra-abdominal and intrathoracic compartment allows bile to enter the pleural space and cause erosion.^3^ There are reports of BBF and biliopleural fistulas within 3 months of LDLT related to early postoperative biliary strictures or leaks.^10,11^ In comparison, our case demonstrates that delayed strictures or persistent leaks may cause a later presentation of BBF.

Typically, the presence of bile (measured with bilirubin) in the sputum is diagnostic of BBF, but it is helpful to locate the site of fistulization to target management.^12^ Diagnostic options include ERCP, bronchoscopy, computed tomography, magnetic resonance cholangiography, and hepatobiliary scintigraphy.^13,14^ Andalkar et al argue that HIDA scans should be the diagnostic test of choice, given their noninvasive approach and ability to visualize small BBFs through functional information.^15^ HIDA was the only approach that visualized our patient's fistula, further supporting its utility.

The multiple management options for BBF include external and internal biliary drainage using image guidance, embolization, or surgical repair.^4^ While surgery was previously preferred, more comprehensive reviews have found less invasive approaches to be a more common first-line intervention if feasible.^1,4,16^

Ammirabile et al suggest a stepwise approach to BBF management that first involves decompression of the biliary system, followed by percutaneous or endobronchial embolization. While more invasive and higher risk, surgery does appear to have a higher success rate and should be considered as a next step for patients with persistent BBFs after embolization attempts.^4^ This patient's BBF resolved without undergoing the risks and potential complications of surgery.

In conclusion, we found postoperative bile leak to be a common risk factor of BBF in LDLT recipients, so clinicians should monitor for biloptysis in these patients. Our case also supports using hepatobiliary scintigraphy, given its increased sensitivity in detecting small BBFs. Finally, we encourage starting with endoscopic or percutaneous biliary decompression before escalation to surgery.

## DISCLOSURES

Author contributions: Each author contributed to the conception and final approval of this manuscript. O. Becker was the primary literature reviewer. O. Becker, D. Suresh, and M. McGrath were the primary manuscript writers. M. McGrath created Figure 1. H. Laird, C. Argo, and N. Shah revised the work. N. Shah is the article guarantor.

Financial disclosure: None to report.

Informed consent was obtained for this case report.

## ABBREVIATIONS:

BBF: bronchobiliary fistula
CT: computed tomography
ERCP: endoscopic retrograde cholangiopancreatography
HIDA: hepatobiliary iminodiacetic acid
IR: interventional radiology
LDLT: living donor liver transplant
MRC: magnetic resonance cholangiography
